# Apatite phosphate doped by cobalt as hight efficient catalyst of multi-component synthesis of therapeutic spiropyrimidine compound

**DOI:** 10.1007/s13659-022-00359-8

**Published:** 2022-09-19

**Authors:** Abdallah Rhihil, Youness Aichi, Mohamed Zahouily, Saïd Sebti, Mohamed El Guendouzi

**Affiliations:** 1 Laboratory of Chemistry Physics Materials and Catalysis (LCPMC), Faculty of Sciences Ben M’sik of Casablanca, Hassan II University of Casablanca, Casablanca, Morocco; 2 Financial Engineering, Governance and Development Laboratory, National School of Business and Management of Casablanca, Hassan II University of Casablanca, Casablanca, Morocco; 3 Laboratory of Materials, Catalysis and Valorization of Natural Resources, Faculty of Sciences and Technology of Mohammedia, Hassan II University of Casablanca, Casablanca, Morocco

**Keywords:** Spiropyrimidine, Natural phosphate, Co/Fap, Heterogeneous catalysis, Multicomponent reaction

## Abstract

Spiropyrimidines vanquish a significant situation in the field of heterocyclic chemistry, they are broadly utilized as an antibacterial, an inhibitor of multidrug opposition, or an antiplatelet and antithrombotic drug. Phosphate, the principal Moroccan mineral wealth occupies a vital spot in the economic sector and its valuation is a goal continually looked for. Among the conceivable outcomes of valorization, its utilization in heterogeneous catalysis which thought about an exceptionally encouraging new way. In this context, the focus on the reaction of the synthesis of spiropyrimidine, catalyzed by natural phosphate (NP) and by fluoroapatite (Fap), are used alone or doped by cobalt. These phosphate catalysts were characterized by XRD, IR and SEM, while the synthesized spiropyrimidine was identified by IR, GC–MS and NMR. The obtained yield with fluorapatite doped by cobalt (Co/Fap), at room temperature in ethanol is very high and shows a high impact performance induced by a synergistic effect compared to that of NP alone.

**Graphical Abstract:**

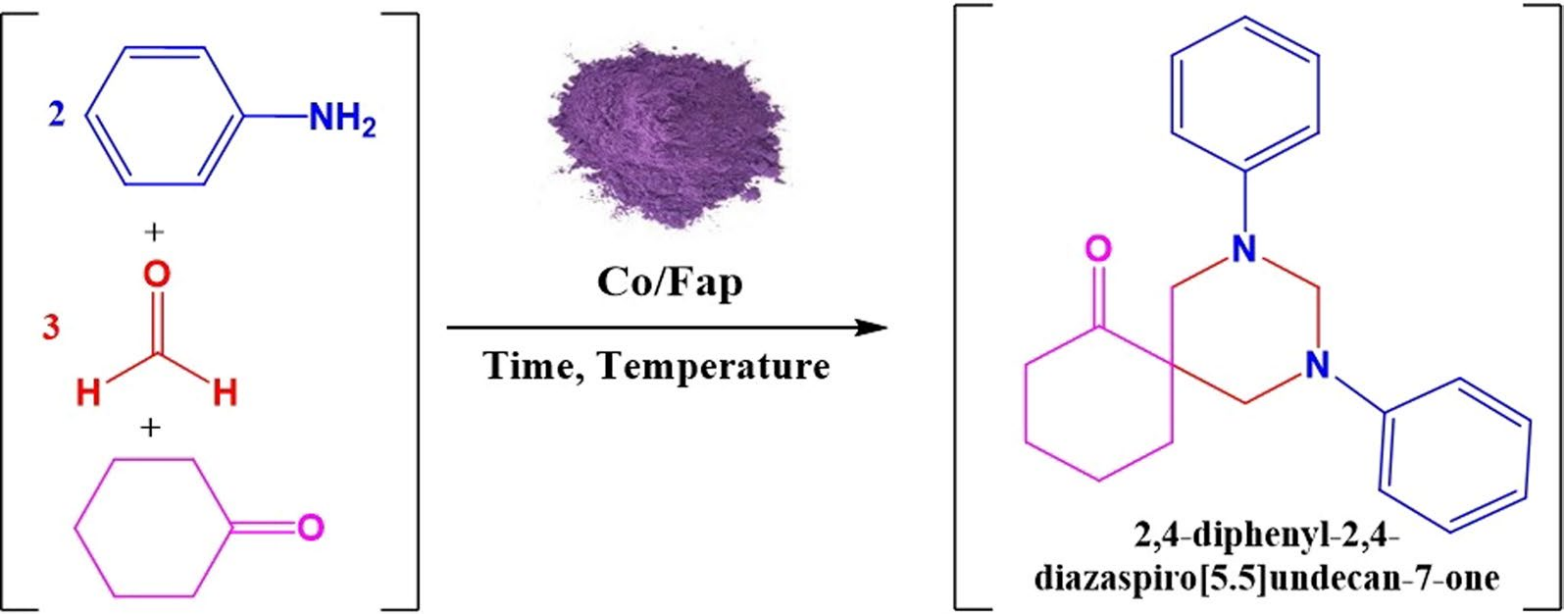

**Supplementary Information:**

The online version contains supplementary material available at 10.1007/s13659-022-00359-8.

## Introduction

In recent years, the chemical industry has experienced a growing need for environmentally friendly chemical processes based on sustainable technologies. This requires a shift from the concept of increasing the economic gains of chemical processes to the concept of eliminating waste at the source and the atom economy. Accordingly, much attention has recently been given to the development of clean techniques for the synthesis of finer products.

Many organic reactions had been catalyst by natural or synthetic’s phosphate such as the Claisen-Schmidt condensation [1], known for the synthesis of chalcones utilized in the treatment of different diseases such as the fight against leukemia cancer. The Suzuki–Miyaura carbon coupling for the synthesis of biphenyls, is used in the therapy of disorders caused by atherosclerosis [2]. The notable Knoevenagel condensation for the preparation of benzylidene malononitrile (BMN) rumored to be powerful inhibitors of tyrosine kinase [3] and the synthesis of α-hydroxyphosphonates by KF/NP [4] and the transposition of α-hydroxyphosphonates by K/NP [5]. These items have organic exercises and drug applications, which had been catalyzed by natural phosphate (NP) [6] or fluoroapatite (Fap) and fluoroapatite alone or doped with metals, showing extremely very high performance.

The spiropyrimidines compounds are the most encountered heterocycles in medicinal chemistry with different antibacterial, antiviral, antitumor and anti-inflammatory applications. This synthesis is carried out by catalysis, a method used in organic chemistry transformations. It has known a very considerable development in the field of scientific research, more particularly heterogeneous catalysis, due to its ease of use, the reuse of the catalyst and its recovery.

The synthesis of biologically active heterocyclic products, by the catalysis of the multicomponent reaction is a powerful strategy to obtain spiropyrimidines in the presence of a solid catalyst by fluoroapatite doped with cobalt (Co/Fap).

N-substituted hexahydropyrimidines are spermidine-nitroimidazole drugs for the treatment of lung carcinoma A549 [7]. N-(4-Aminobutyl) hexahydropyrimidine and N-(3-aminopropyl) hexahydropyrimidine have been shown to compete with spermidine for uptake by L1210 cells, as well as the conditions for optimal growth in suspension culture [8]. They form structural units in trypanothione reductase inhibitory ligands for the regulation of oxidative stress in parasitic cells [9], due to their significant biological activity, hexahydropyrimidines have received attention in recent years.

### Preparation of the fluorapatite doped by cobalt (Co/Fap) catalyst

Fluorapatite doped by cobalt (Co/FAP) was elaborated by co-precipitation, following a similar process as fluorapatite (Fap) [10].

The Ca_8_Co_2_(PO_4_)_6_F_2_ is prepared under the temperature of 80 °C, flack 7.93 g of (NH_4_)_2_HPO_4_, 70 mL of NH_4_(OH), 1 g of NH_4_F in 250 mL ultra-pure water, and adding on the mixture 18.89 g of Ca(NO_3_)_2_ and 5.82 g of Co(NO_3_)_2_6H_2_O dissolved in 150 mL of ultra-pure water. The mixture was heating for 3 h at pH 9, the formed powder is washed with ultra-pure water and dried for 24 h at 150 °C and then calcined at 600 °C for 2 h.

### Characterization of the catalyst

**Fig. 1:**
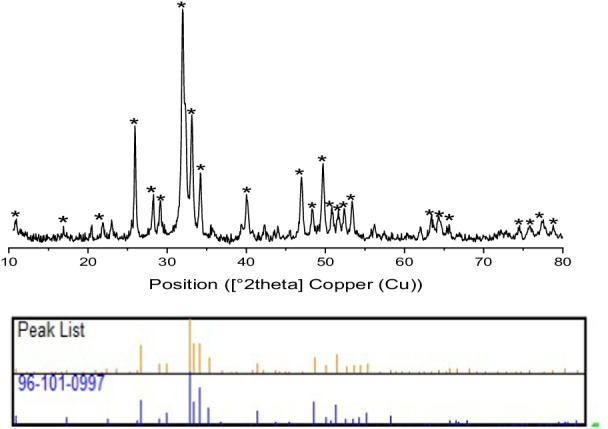
X-ray diffraction patterns of Co/Fap

The diffractogram of fluorapatite doped by cobalt Ca_(10–2)_Co_2_(PO_4_)_6_F_2_ is identified as that of the fluorapatite [7]. The outcomes are given underneath in the accompanying Fig. 1.

The X-ray diffraction pattern of the Co/Fap synthesis was highly selective where cobalt is incorporated in the structure. There is an absence of impurities such as calcium fluoride or phosphate, and cobalt or calcium oxide. Indeed, the X-ray diffraction pattern of the calcined Co/Fap showed the appearance of a single solid phase which is similar to the fluorapatite (Ca_10_(PO_4_)_6_F_2_) [11].

**Fig. 2:**
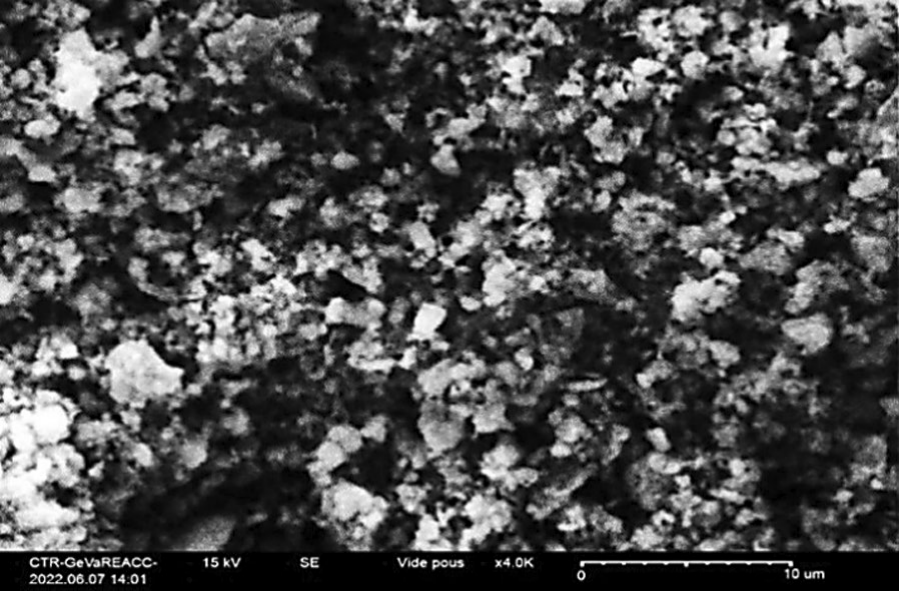
SEM imaging of fluorapatite doped by cobalt

The analysis of fluorapatite doped by cobalt using Scanning Electron Microscope (SEM) shows that the material agglomerates with a size under 2 µm (Fig. 2).

The IR spectrum of Co/Fap shows bands at 556 and 607 cm^−1^ connected with the PO_4_^3−^ moiety as well as new groups at 2850 and 2926 cm^−1^ that didn’t exist in the Co/Fap IR range. This implies that cobalt is very much embedded in the fluorapatite structure. Table 1 shows the IR retention groups of Co/Fap.

**Table 1:** Infrared bands of Co/Fap

Bands position (cm^−1^)	Assignment
556	PO_4_^3−^
607	PO_4_^3−^
2850	Co–O–P
2926	Co–O–P

### Procedure for the synthesis of spiropyrimidine and catalysts effect

**Scheme 1:**
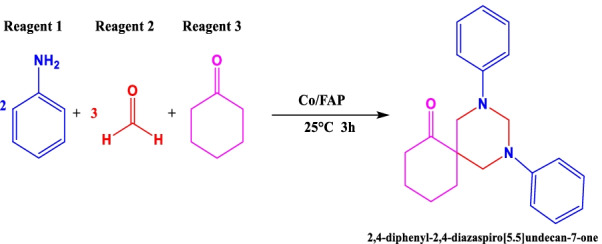
Multicomponent reaction for the synthesis of spiropyrimidine

Multi-component reactions allow getting objective molecules from more than two reagents. One of them is the multicomponent reaction for the synthesis of spiropyrimidines (Scheme 1).

**Fig. 3:**
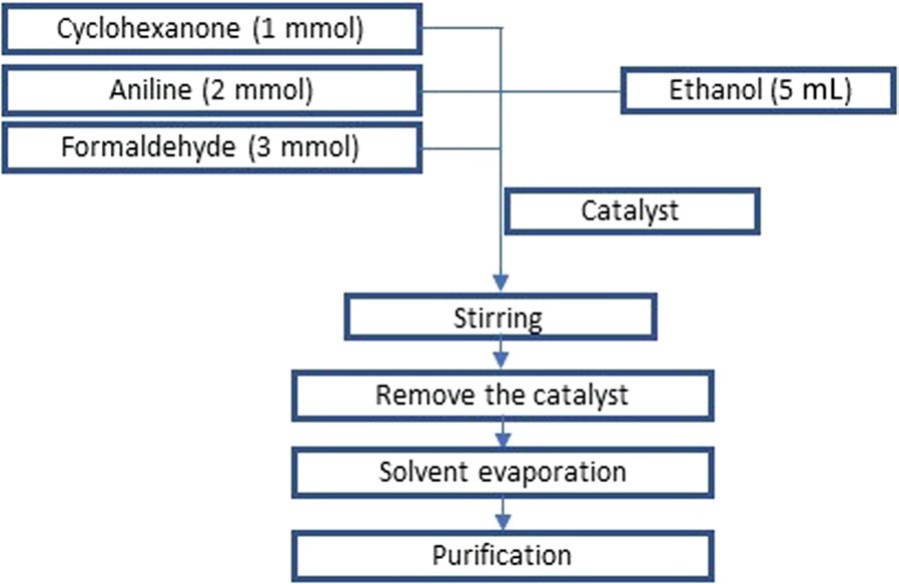
Spiropyrimidine synthesis reaction procedure

**Fig. 4:**
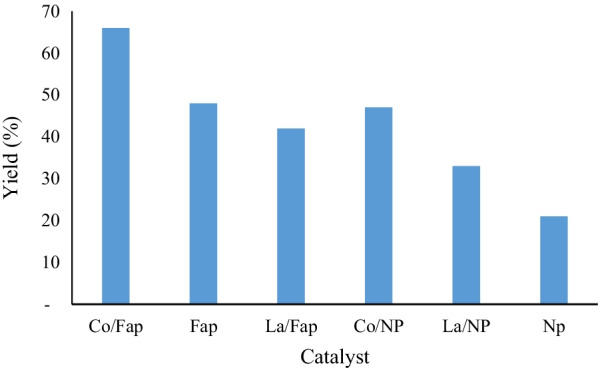
Synthesis of spiropyrimidine by different catalysts

In this work, various tests were performed to examine the effectiveness of Co/Fap for catalysis of the multicomponent reaction of spiropyrimidine, while following the procedure (Fig. 3):

In 25 mL boiling flasks the three beginning reagents: 2 mmol aniline, 1 mmol cyclohexanone and 3 mmol formaldehyde was mixed, 5 mL of ethanol as solvent had been added, the reaction blend was mixed at room temperature for 3 h. At the end of the reaction, the mixture was isolated from the catalyst by vacuum filtration, the solvent was evaporated by rotavapor, and the product item was gotten is purified by recrystallization. Several heterogeneous catalysts were tested in the multicomponent reaction of synthesis. The results are presented in Fig. 4.

In the wake of testing several solid catalysts in this reaction, including natural phosphate (NP), NP doped with lanthanum (La/NP), NP doped with cobalt (Co/NP), fluorapatite (Fap), Fap doped with lanthanum (La/Fap) and Fap doped with cobalt (Co/Fap), we noticed that Co/Fap gave the best yield (66%), and NP alone gave a yield of 21%, while without a catalyst, the reaction is inactive.

The speroperimidine 2,4-bis-phenyl-2,4-diazaspiro [5.5]undecane-7-one obtained was identified by gas chromatography GC–MS and characterized by IR, ^1^H NMR and UV.

- *IR spectrum: Band in 1597 cm*^*−1*^* which correspond to the bond C*=*O, bands in 1597 and 1499 cm*^*−1*^* which correspond to the bond C*=*C of the aromatic, bands at 3034,749 and 696 cm*^*−1*^* relating to the bond* = *C–H, as well as the band in 1167 and 937 cm*^*−1*^* corresponding to the bond C–C and finally of the bands in 1226 and 1334 cm*^*−1*^* corresponding to the bond C–N.*
- ^*1*^*H NMR spectrum (400 MHz, CDCl*_*3*_*): δ 1.58–1.63 (m, 2H), 1.83–1.95 (m, 4H), 2.45 (t, 2H), 3.46 (d, 2H), 3.63 (d, 2H), 4.09 (d, 1H), 4.66 (d, 1H), 6.79–7.23 (m, 10H, Ar-Region).*
- ^*13*^*C NMR (100 MHz, CDCl*_*3*_*): 198.13, 149.65, 129.33, 120.21, 118.49, 68.21, 55.11, 50.73, 39.78, 34.36, 27.73, 20.33.*
- *GC–MS: Column: DB-5 (30 m* × *0.25 mm i.d.* × *0.25 µm film thickness); carrier gas: Helium, 1 mL/min; MS transfer line: 325 °C; solvent delay: 6.5 min. MS mass spectrum m/z: 321.1 (M*^+^ + *1).*
- *UV spectrum shows adsorption at the wavelength λ*_*Max*_ = *239 nm.*

After the preliminary study which included the catalysts study with the same operative conduction and an amount of 20 mg, it was found that fluorapatite doped by cobalt is a good catalyst for the multicomponent reaction of spiropyrimidine synthesis.

### Mass and solvent effects

**Fig. 5:**
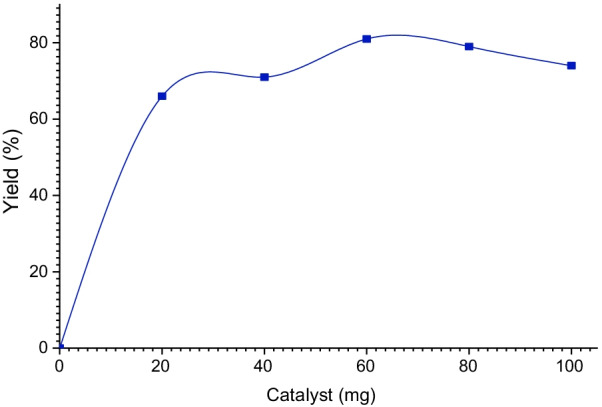
Influence of the catalyst mass Co/Fap on Yield

The study of the mass impact of catalyst uses in the multicomponent spiropyrimidine reaction is performed using successively 20, 40, 60, 80, and 100 mg of the Co/Fap catalyst, with following the procedure described above (Fig. 3). The results are regrouped in Fig. 5.

From the obtained results, it can be seen that 60 mg of the catalyst is the sufficient amount to obtain yield of 81%, beyond this amount, the yield drops to 74%.

**Fig. 6:**
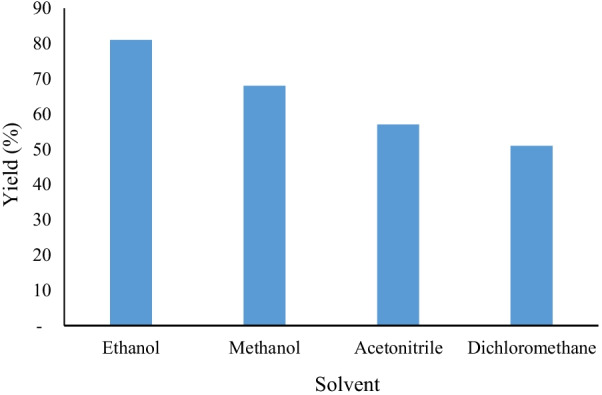
Influence of solvents on yield

The solvent is an important factor to promote contact between the components of the reaction mixture. To optimize this factor, several solvents have been used while keeping the same conditions as before. The obtained results are shown in Fig. 6.

It is noticed that ethanol gave a yield of 81% compared to acetonitrile and dichloromethane which gave a relatively low yield of 57% and 51% respectively (Fig. 6). While in the presence of water or without solvent, the reaction is practically inactive.

### Kinetic study of the reaction and temperature effect

**Fig. 7:**
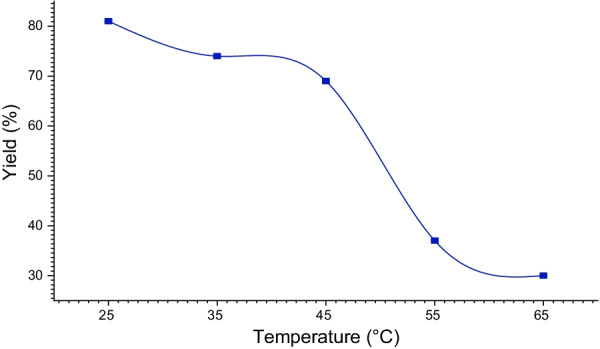
Influence of temperature on the yield

The yield of the reaction was performed at various temperatures from 25 °C to 65 °C (Fig. 7). It is noted that by increasing the temperature, the reaction yield decreases to 30% at 65 °C, while the best yield 81% is obtained at room temperature.

**Fig. 8:**
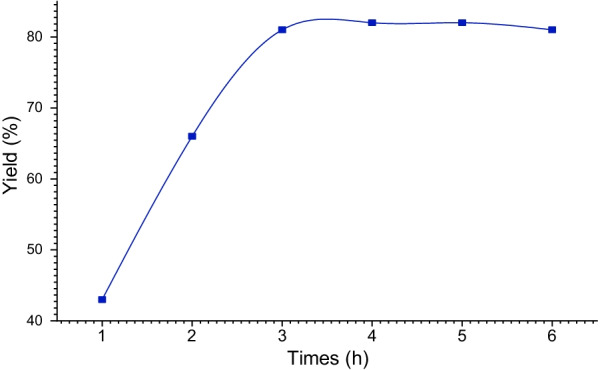
Influence of the reaction time on the yield

The reaction time was improved, while keeping the same experimental conditions, in the presence of Co/Fap. The synthesis of spiropyrimidine was performed under the same conditions as before and at room temperature of the reaction medium. The increase in the yield of the reaction was observed at 3 h of the reaction time, beyond that, a stabilization of the yields was reached (Fig. 8).

## Comparison of the results

The results reported in the literature concerning the operating conditions for the synthesis of spiropyrimidine and the yields of the reactions obtained by different catalysts, in comparison with the results obtained by Co/Fap, are grouped in Table 2.

**Table 2:** Comparison of the spiropyrimisdine synthesis reaction by different catalysts

Reagent 1	Catalysts/Solvent	Type of catalysis	T (°C)	Time (h)	Yield (%)	References
Aniline	Co/ Fap 10 mol % (50 mg)/ethanol (5 mL)	A	25	3	81	This work
p-tolylamine	Lactic acid/DMSO	B	25	3–5	80	[12]
4-Fluoroaniline	CuFe_2_O_4_ 10 mol %/ethanol (5 mL)	A	25	3	82	[13]
4-Chloroaniline	CuFe_2_O_4_10 mol %/ethanol (5 mL)	A	25	3	79	[13]
4-Chloroaniline	In(OTf)_3_ 10 mol %/CH_2_Cl_2_	B	25	4	78	[14]
Aniline	(H_14_[NaP_5_W_30_O_110_])/SiO_2_ 10 mol %/DMSO (5 mL)	A	25	20	60	[15]
Aniline	(H_14_[NaP_5_W_30_O_110_]) /SiO_2_ 10 mol %/DMSO (5 mL)	A	SF	20	60	[15]
Aniline	Dy(III)/Chitosan (100 mg)/H_2_O (5 mL)	A	25	50 min	87	[16]
Aniline	Trifluoroethanol (5 mL)	B	25	3	84	[17]
Aniline	(S)-proline 20 mol %/DMSO (5 mL)	B	25	30	53	[18]

*SF* solvent reflux, *A* heterogeneous media, *B* homogeneous media

The comparison of the yields of spiropyrimidine synthesis, under the reaction operating conditions, in the presence of different catalysts, in a heterogeneous (category A) and homogeneous (category B) media, reported in the literature, with that obtained with fluorapatite doped by cobalt, shows that our catalyst can be considered among the most efficient catalysts, next to Dy(III)/Chitosan [16], taking into consideration the amount of catalyst used, the reaction temperature and the required advantages of the type of reaction media, in other words catalysis in a heterogeneous solid/liquid medium. So, Co/Fap allows to have a yield of 81%, at room temperature, a reaction time not exceeding 3 h and with only 50 mg of the catalyst.

The obtained results are very interesting and open another way to explore this phosphate catalyst in the synthesis of other compounds biologically active, and respecting the environment and the principles of green chemistry.

## Conclusion

Given the importance of spiropyrimidine products and their biological activities as well as its applications in the medical field in chemotherapy as anti-cancer in the fight against lung carcinoma A549, against L1210 cells and leukemia, as well as for the regulation of oxidative stress in parasitic cells, in the context of environmental protection and clean chemical synthesis, the focused in heterogenous catalysis as one of the fundamental pillars of green chemistry, which aims at atoms saving and reducing the chemical impact on the environment.

The heterogeneous catalysis by phosphates has had a great development in several chemical processes, in particular in organic synthesis, with interesting catalytic efficiency, thanks to the facility of its preparation, its great reactivity and selectivity and its abundance, offering a great advantage compared to homogeneous catalysis. NP have both acidic and/or basic properties, structure flexibility, capacity to disperse a catalytic active phase, and low cost.

The focused is on the optimization of the spiropyrimidine synthesis reaction, studying the effects of influencing parameters on the reaction yield. From the results of the tests performed, a maximum yield of 81% was obtained using ethanol as solvent, with 60 mg of catalyst Co/Fap, working at room temperature and with a reaction time of 3 h.

This work allowed us to show the effectiveness of the Co/Fap catalyst as an efficient catalyst for this type of reaction, and opens the perspective to make further studies generalize this synthesis (Additional file 1).

## Supplementary Information

**Additional file 1.** Additional analysis and characterization.
