# Supplementary material for: Apatite phosphate doped by cobalt as hight efficient catalyst of multi-component synthesis of therapeutic spiropyrimidine compound
Source: Nat Prod Bioprospect. 2022 Sep 19;12(1):35. doi: 10.1007/s13659-022-00359-8 (PMC9485353; doi:10.1007/s13659-022-00359-8)
Supplement: Supplementary file 1 — Additional file 1. Additional analysis and characterization. [file 13659_2022_359_MOESM1_ESM.pdf]

Supplementary Information

Apatite phosphate doped by cobalt as hight efficient catalyst of multi-component synthesis of therapeutic Spiropyrimidine compound

Authors : Abdallah RHIHIL[2]*, Youness AICHI[1], Mohamed ZAHOUILY[3] Saïd SEBTI[1], Mohamed EL GUENDOUZI[1]

Affiliation :

[1]*Laboratory of Chemistry Physics Materials & Catalysis, Faculty of Sciences Ben M'sik of Casablanca, Hassan II University of Casablanca (Morocco)*

[2]*Financial Engineering, Governance and Development Laboratory, National School of Business and Management of Casablanca, Hassan II University of Casablanca (Morocco)*

Table of content

1. X-ray Diffractogram of Co/Fap 2
2. Scanning Electron Microscope (SEM) of fluorapatite doped by cobalt 2
3. The IR spectrum of Co/Fap 3
4. The IR spectrum of synthesized compound 3
5. NMR Spectrum of synthesized compound 4
6. GC chromatogram of synthesized compound 5
7. Mass spectrum of synthesized compound 5
8. UV spectrum of synthesized compound 5

1. **X-ray Diffractogram of Co/Fap**

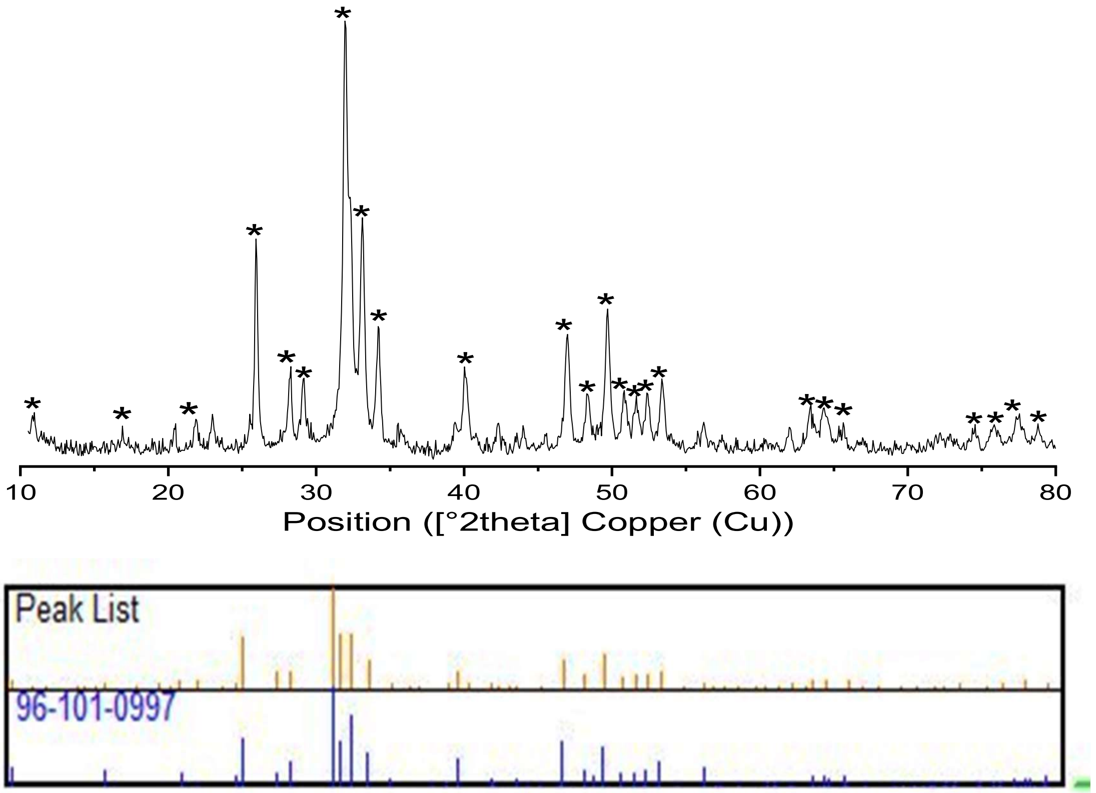

Fig. 1: X-ray diffraction patterns of Co/Fap

2. **Scanning Electron Microscope (SEM) of fluorapatite doped by cobalt**

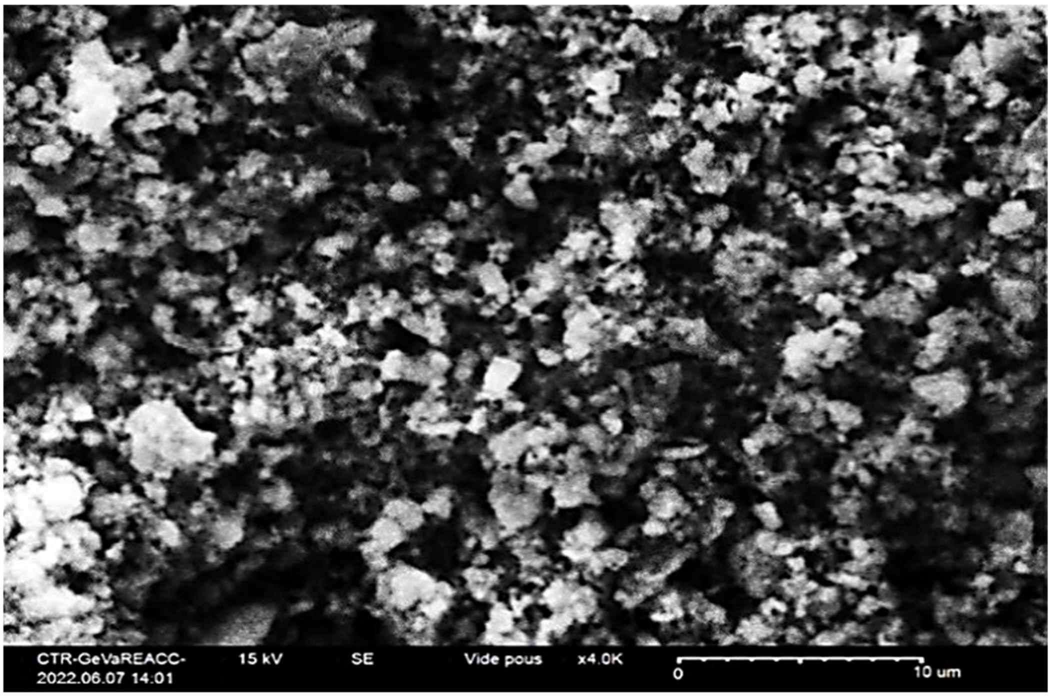

Fig. 2: SEM imaging of fluorapatite doped by cobalt

3. The IR spectrum of Co/Fap

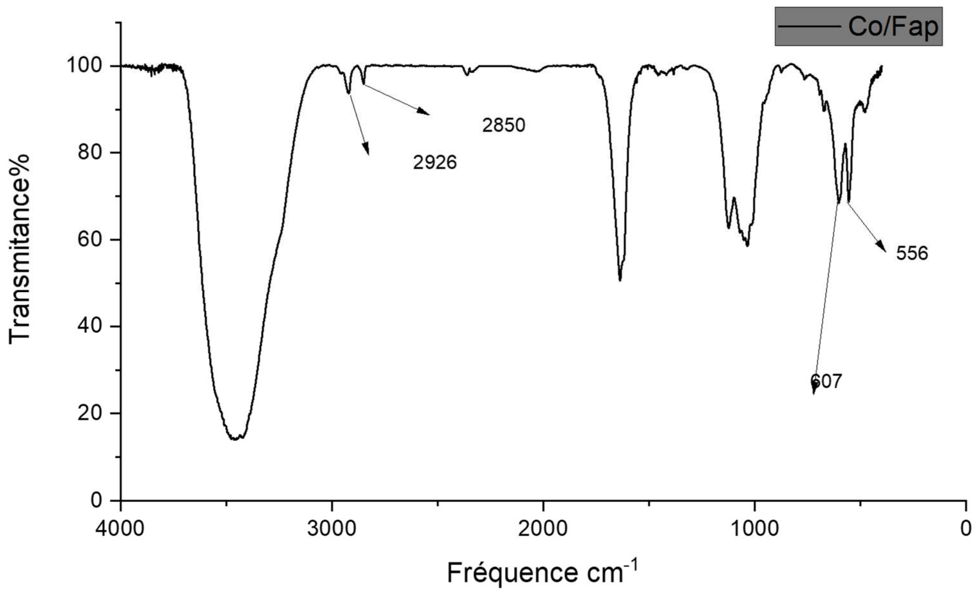

Table 1: Infrared bands of Co/Fap

Bands position (cm^{-1})	Assignment
556	PO_4^{3-}
607	PO_4^{3-}
2850	Co-O-P
2926	Co-O-P

4. The IR spectrum of synthesized compound

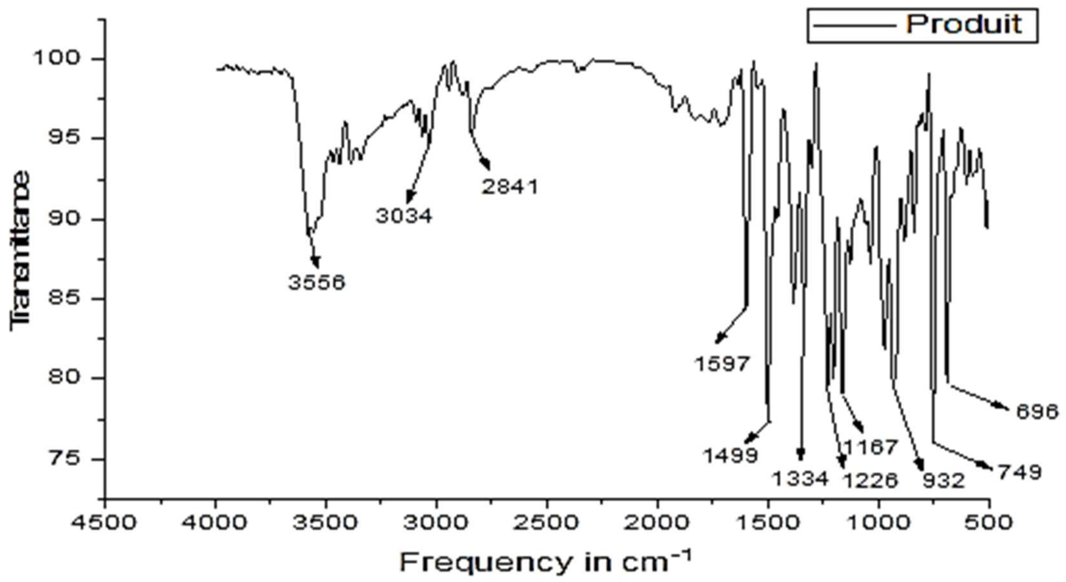

Infrared Spectra of 2,4-Bis-phenyl-2,4-diazaspiro[5.5]undecan-7-one

5. **NMR Spectrum of synthesized compound**

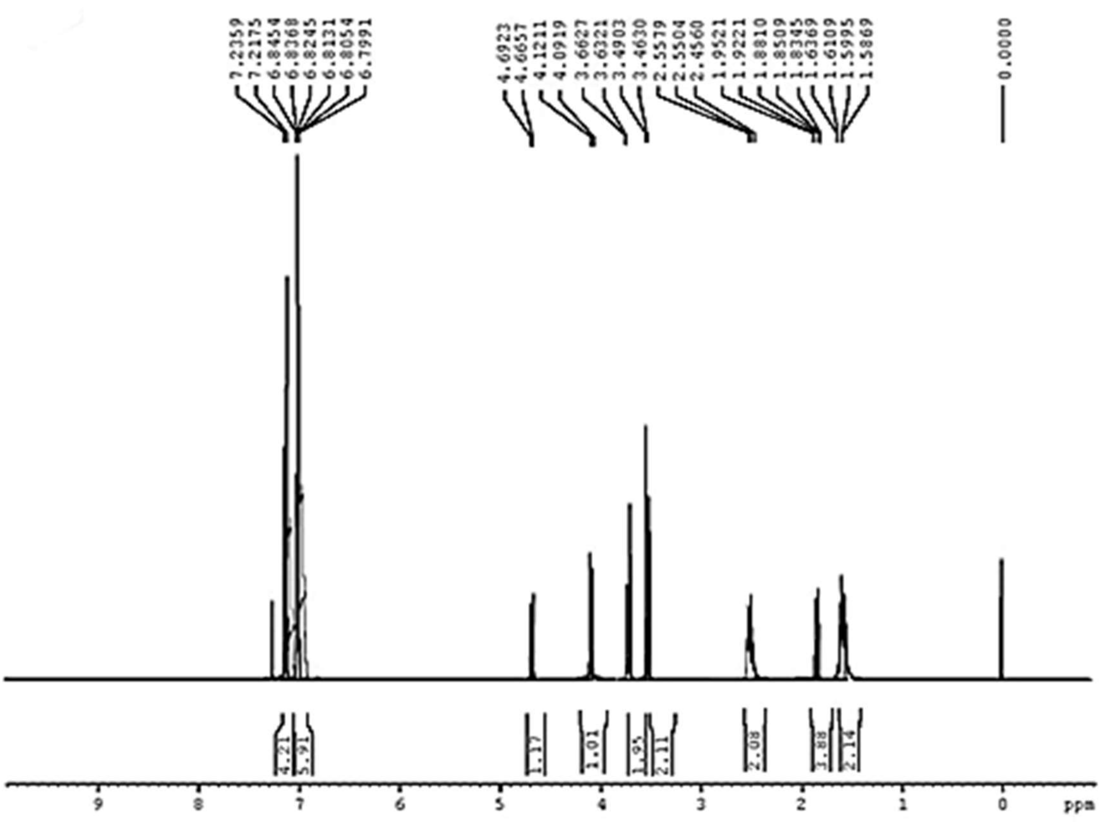

^{1}H-NMR Spectra of 2,4-Bis-phenyl-2,4-diazaspiro[5.5]undecan-7-one

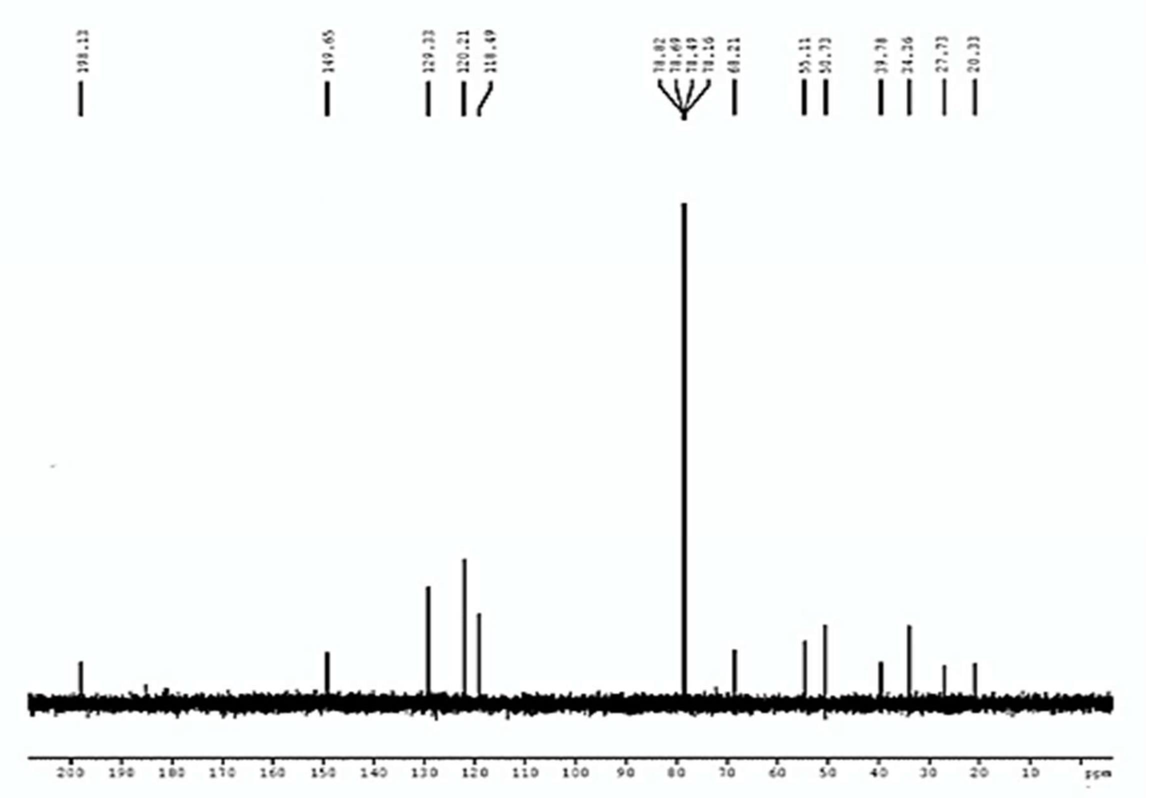

^{13}C-NMR Spectra of 2,4-Bis-phenyl-2,4-diazaspiro[5.5]undecan-7-one

6. GC chromatogram of synthesized compound

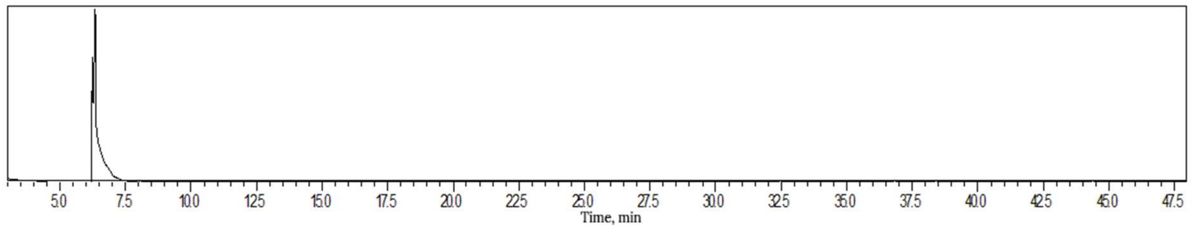

GC chromatogram of 2,4-Bis-phenyl-2,4-diazaspiro[5.5]undecan-7-one

7. Mass spectrum of synthesized compound

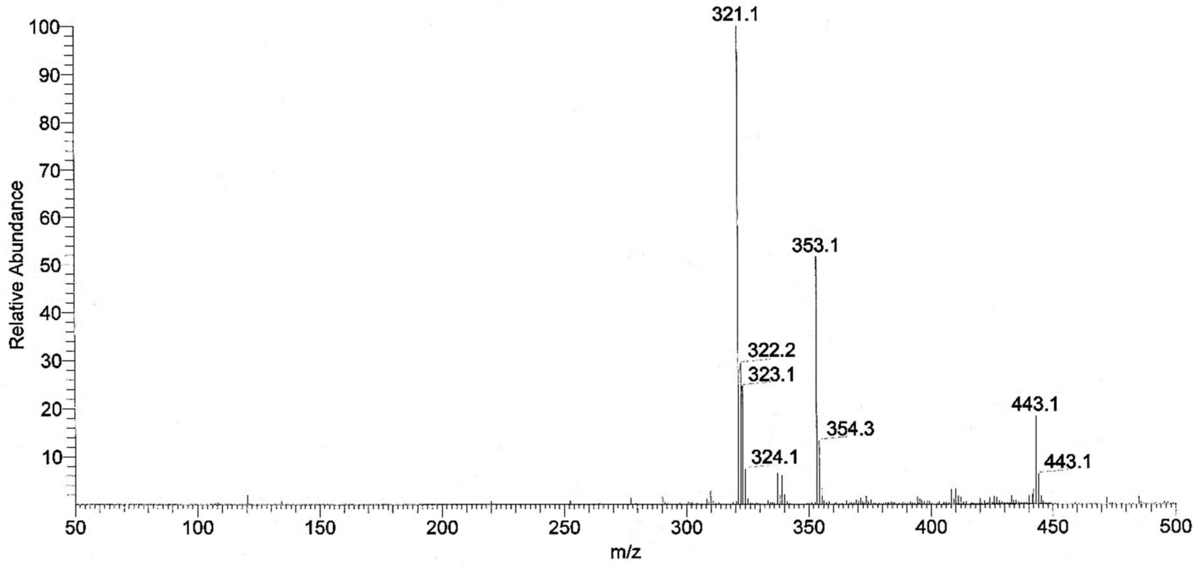

Mass spectrum of 2,4-Bis-phenyl-2,4-diazaspiro[5.5]undecan-7-one

8. UV spectrum of synthesized compound

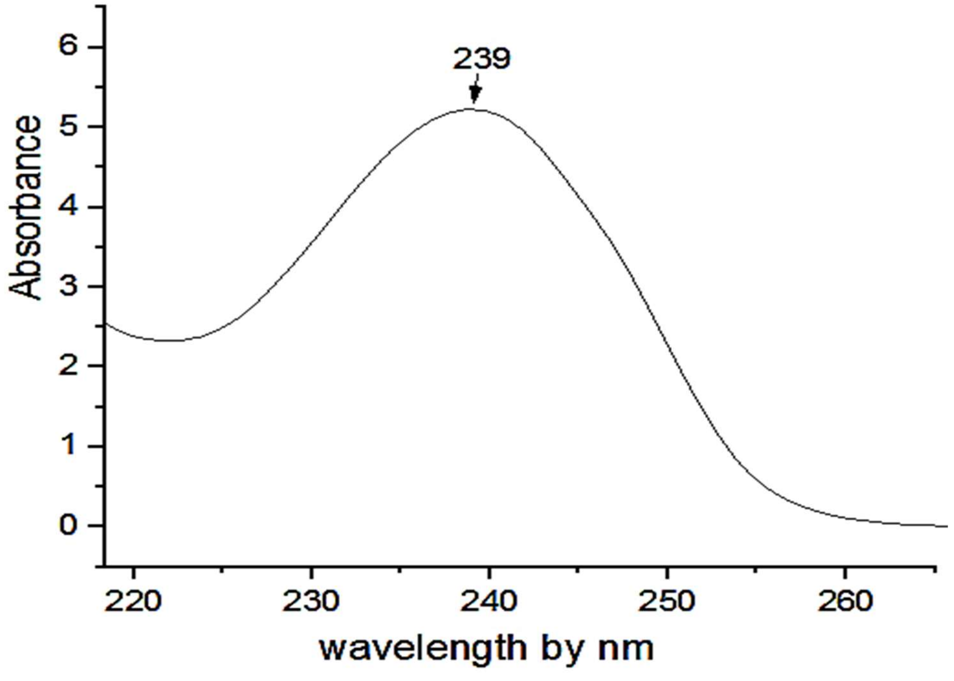

UV spectrum of 2,4-Bis-phenyl-2,4-diazaspiro[5.5]undecan-7-one
